# Short-Time β-Alanine Supplementation on the Acute Strength Performance after High-Intensity Intermittent Exercise in Recreationally Trained Men

**DOI:** 10.3390/sports7050108

**Published:** 2019-05-09

**Authors:** Marcelo Conrado Freitas, Jason Cholewa, Valéria Panissa, Giovanni Quizzini, João Vitor de Oliveira, Caique Figueiredo, Luis Alberto Gobbo, Erico Caperuto, Nelo Eidy Zanchi, Fabio Lira, Fabrício Eduardo Rossi

**Affiliations:** 1 Department of Nutrition, University of Western São Paulo (UNOESTE), Presidente Prudente, SP 19050-920, Brazil; marceloconrado013@gmail.com; 2 Department of Kinesiology, Coastal Carolina University, Conway, SC 29576, USA; jcholewa@coastal.edu; 3 Department of Sport, School of Physical Education and Sport, University of São Paulo, São Paulo, SP 05508-030, Brazil; valeriapanissa@gmail.com; 4 Skeletal Muscle Assessment Laboratory, School of Technology and Sciences, Department of Physical Education, São Paulo State University, Presidente Prudente, SP 19060-900, Brazil; giovanniquizzini@gmail.com (G.Q.); luis.gobbo@unesp.br (L.A.G.); 5 Exercise and Immunometabolism Research Group, Department of Physical Education, São Paulo State University (UNESP), Presidente Prudente, SP 19060-900, Brazil; joaonevesspa@gmail.com (J.V.d.O.); caiquefigueiredo22@gmail.com (C.F.); fabio.lira@unesp.br (F.L.); 6 University São Judas Tadeu, São Paulo, SP 05503-001, Brazil; ericocaperuto@gmail.com; 7 Laboratory of Cellular and Molecular Biology of Skeletal Muscle (LABCEMME), Department of Physical Education, Federal University of Maranhão, São Luís, MA 65080-805, Brazil; neloz@ig.com.br; 8 Immunometabolism of Skeletal Muscle and Exercise Research Group, Department of Physical Education, Federal University of Piauí (UFPI), Teresina, PI 64049-550, Brazil; 9 Associate Graduate Program in Health Science, Federal University of Piauí (UFPI), Teresina, PI 64049-550, Brazil

**Keywords:** ergogenic aids, strength, amino acids, pre-workout, body composition

## Abstract

(1) Background: We investigated the effects of 28 days of beta-alanine (β-alanine) supplementation on the acute interference effect of high-intensity intermittent exercise (HIIE) on lower-body resistance exercise performance, body composition, and strength when combined with a resistance training program. (2) Methods: Twenty-two males were randomized into: β-alanine supplementation (6.4 g/day) or placebo (6.4 g/day maltodextrin) during 28 days. Total body water, intracellular and extracellular water, fat-free mass (FFM), and fat mass were assessed using bioelectrical impedance. Participants performed 5000-m HIIE (1:1 effort and rest ratio) followed by resistance exercise (four sets of 80% at 45° leg press until muscular failure) at baseline and after 28 days. The resistance training program consisted of three sets of 10 to 12 RM with 90 s of rest, four days per week. (3) Results: For the post-HIIE leg press volume, higher values were observed post-training than pre-training, but no group x time interaction was observed. There was a non-significant trend for an interaction in the FFM change (β-alanine = 2.8% versus placebo = 1.0%, *p* = 0.072). (4) Conclusion: Twenty-eight days of β-alanine supplementation did not prevent acute strength loss during resistance exercise after high-intensity interval exercise, nor increase strength or hypertrophic adaptations associated with resistance training.

## 1. Introduction

Concurrent exercise describes performing both resistance and aerobic or high-intensity intermittent exercise (HIIE) during the same training session, and a growing body of literature suggests that acute HIIE impairs subsequent resistance exercise performance [1,2,3,4]. The acute interference effects have been associated with reductions in muscular strength and lower total volume (repetitions x load) in response to central and peripheral fatigue accumulation induced by HIIE [4,5,6]. Leveritt and Abernethy [7] suggested intracellular acidosis as a possible factor that contributes to reduced strength performance when HIIE is performed prior to resistance exercise. On the other hand, Abernethy [8] reported a reduction in force output 4 h following an intense aerobic bout of exercise. Although not measured, muscle pH likely returned to baseline values 4 h after exercise, suggesting that mechanisms other than muscle acid–base balance, such as central fatigue or the acute immunological response, may also contribute to impaired acute strength performance [2,8]. In this sense, sport supplement interventions that attenuate peripheral fatigue may be an effective strategy to reduce the negative effects of acute HIIE prior to resistance exercise [9,10,11].

In the last decade, several studies have demonstrated that beta-alanine (β-alanine) supplementation increases the intramuscular buffer carnosine, thereby enhancing intramuscular hydrogen ion (H^+^) buffering and calcium sensitivity [12,13], and attenuating reductions in pH following intense exercise [14]. If a threshold level of muscle acidosis as a result of HIIE does indeed compromise subsequent resistance exercise performance, then increasing buffering capacity with β-alanine supplementation may prevent muscular force decay, and thereby attenuate the acute interference effect. While chronic β-alanine supplementation has been used to improve acute performance in exercise tasks that rely on rapid glycolytic metabolism [15], to our knowledge, the interaction between β-alanine supplementation and the acute interference of HIIE on subsequent resistance exercise performance has yet to be investigated.

With an increasing energy output capacity, resistance trainers may perform more volume and a larger amount of muscle metabolites may be produced, thereby promoting cellular swelling and potentiating hypertrophy [16]. β-alanine supplementation has been shown to increase muscle performance during resistance training, and such increases have occurred together with increases in lean mass [17]. Kern et al. [17] found that eight weeks of β-alanine supplementation (4 g/day) increased lean body mass and performance in collegiate wrestlers and football players. On the other hand, Outlaw et al. [18] showed that eight weeks of β-alanine supplementation (3.4 g/day for eight weeks) combined with resistance training (RT) improved lower-body muscular endurance, but did not influence maximal strength, lean body mass, fat mass, and percent body fat in collegiate women. Kendrick et al. [19] verified the effects of β-alanine supplementation with 6.4 g per day during four weeks of isokinetic training with the right leg, whilst the left leg was untrained and muscle biopsies were taken from the vastus lateralis immediately before and at the end of the supplementation period. Kresta et al. [20] analyzed the effects of 28 days of β-alanine and creatine supplementation on muscle carnosine, body composition, and exercise performance in recreationally active females. However, both studies reported no improvements in fat-free mass and fat mass, respectively [19,20]. While β-alanine supplementation appears to be an effective ergogenic aid in high-intensity tasks that require significant strength endurance, its ability to enhance hypertrophic adaptations during resistance training remains ambiguous.

The primary purpose of this study was to investigate the effects of 28 days of β-alanine supplementation on the acute interference of HIIE on lower body resistance exercise performance. In addition, given the conflicting reports related to β-alanine supplementation and muscle hypertrophy, our second aim was to investigate changes in lean mass with β-alanine supplementation during resistance training. Considering that chronic supplementation is necessary to increase carnosine in muscle tissue [21,22], and to control for training adaptations, all the participants of the study performed a supervised progressive resistance training program concomitant with a period of 28 days of supplementation.

We hypothesize that 28 days of β-alanine supplementation will attenuate the loss of strength performance observed after acute HIIE. We also hypothesize that β-alanine supplementation will induce greater resistance training volumes and thereby inducing greater increases in lean mass than placebo.

## 2. Materials and Methods

### 2.1. Subjects

Twenty-four recreationally resistance trained males (age = 23.7 ± 3.9 years; body mass = 78.6 ± 12.0 kg; height = 177.3 ± 5.5 cm) with at least six months of resistance training experience (experience: 4.0 ± 1.4 years, weekly training frequency: four to five days, training duration: 40–60 min) were recruited for this study. During the 28 days of intervention, one subject from the placebo dropped out of the study due to personal/family problems and was excluded; therefore, the treatment groups consisted of β-alanine supplementation (*n* = 12) and placebo (*n* = 11).

The inclusion criteria were: (1) age between 20–30 years; (2) participation in periodized strength training ≥6 months; (3) had not used any dietary supplement, ergogenic substance, or medicine for at least three years prior to the study; (4) no smoking or alcohol consumption; (5) no contraindications involving the cardiovascular system, muscles, joints, or bones of the lower limbs that could limit exercise. The study was approved by the Ethics Research Group of the University of São Judas, São Paulo-SP, Brazil (Protocol number: 66523717.2.0000.0089), and the research was conducted according to the 2013 Revision of the Declaration of Helsinki. All the participants signed a consent form and they were informed about the purpose of the study and the possible risks.

### 2.2. Experimental Design

**Figure 1:**
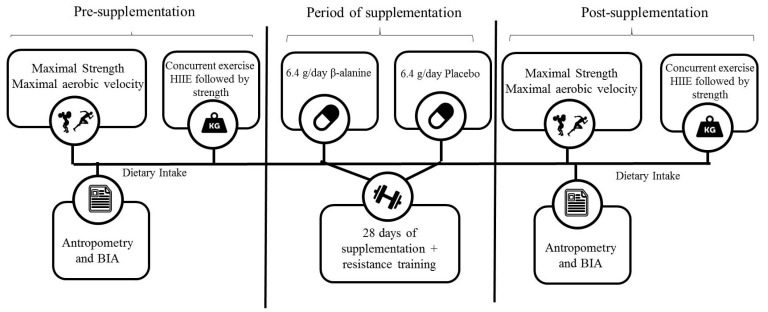
Experimental Design.

This study used a randomized, double-blind design. Participants were paired based on initial one-repetition maximum test (1 RM) and the maximal aerobic velocity (*v*VO_2max_) reached on the incremental test and then randomly assigned to one of two treatment groups: placebo or β-alanine. On the first visit, participants were assessed for anthropometrics and body composition. During the second, third, and fourth visit, the participants completed a familiarization with the exercise routines, and on the following fifth and sixth visit, the maximal aerobic velocity test and 1 RM were performed separated by 96 h. On the seventh visit, the concurrent exercise session was performed. Afterwards, the participants completed 28 days of supplementation plus resistance training and returned to the laboratory on the 29th day to repeat the body composition assessment, the maximal aerobic velocity test, and 1 RM. After this, the concurrent exercise session was performed one week later. The total number of repetitions performed was recorded for each set. Blood lactate concentration was obtained at rest, during exercise (half of session), and 5 min, 10 min, and 30 min after exercise. The subjective rating of perceived exertion was recorded at the end of exercise. The experimental design is illustrated in Figure 1.

### 2.3. Procedures

#### 2.3.1. Maximal Aerobic Velocity and One-Maximum Repetition Test

Initially, the participants completed three familiarization sessions to become acquainted with incremental test and 1 RM test procedures. The subjects performed an incremental test to volitional exhaustion. The initial treadmill (Inbramed, modelo MASTER CI, Brazil) speed was set at 8.0 km/h and increased by 1 km/h per 2-min stage until the participant could no longer continue. The maximal velocity reached in the test was defined as the velocity at VO_2max_ (*v*VO_2max_). When the subject was not able to finish the 1-min stage, the speed was expressed according to the time of permanence in the last stage, which was determined as the following: *v*VO_2max_ = velocity of penultimate stage + [(time, in seconds, remained at the last stage multiplied by 1 km/h)/60 s] [23].

For the one-maximum repetition test (1 RM), a warm-up was performed on a treadmill for 5 min at 50% of velocity at *v*VO_2max_ prior to testing, and one subsequent set of 10 repetitions at approximately 50% of the 1 RM. The load was increased gradually (10–15%) during the test until the participants were no longer able to perform the entire movement, and three to five attempts were allowed. The one-repetition maximum test (1 RM) was conducted to determine the maximum strength using a 45° leg press (Ipiranga^®^, São Paulo, SP, Brazil). Then, the participants had up to five attempts to achieve the 1 RM load with a 3 to 5-min interval between trials, according to Rossi et al. [24].

#### 2.3.2. Acute Concurrent Exercise Session

Concurrent exercise testing was conducted pre- and post-intervention. Participants performed a warm-up at 50% of *v*VO_2max_ for 5 min, and after a 2-min rest interval, the exercise bout was started. The endurance exercise consisted of a five-kilometer intermittent run on a treadmill, corresponding to 1 min at *v*VO_2max_ followed by 1 min of passive recovery (1:1 work-to-rest ratio). Subjects performed 23 ± two sprints, and all the subjects were able to maintain the pace for the five kilometers. This exercise protocol was implemented previously by de Salles Painelli et al. [9] and de Souza et al. [1], who reported significantly reduced lower body strength after a five-kilometer high-intensity intermittent run. After a 10-min passive rest interval, the subjects performed four sets at 80% 1 RM in the 45° Leg press exercise (Ipiranga^®^, Presidente Prudente, SP, Brazil) to concentric failure, and each set was separated by a 2-min rest interval. The maximum number of repetitions performed was recorded for each set.

#### 2.3.3. Resistance Training Protocol

One week after the concurrent exercise session, subjects performed 28 days of a progressive resistance training program (Table S1, Supplementary Material). Initially, the participants performed a warm-up at 50% of *v*VO_2max_ for 5 min on a treadmill and 10 repetitions at approximately 50% of the 1 RM during the first set. The resistance training program consisted of three sets of 10 to 12 RM with 90 to 120 s of rest between sets. The sets were carried out until transient concentric muscular failure, and the load was adjusted for each exercise as needed on successive sets to ensure that the subjects achieved failure in the target repetition range (10 to 12 maximum repetitions). The cadence of repetitions was performed with a concentric action of approximately one second, an eccentric action of approximately two seconds, and a one-second pause between each repetition. The participants were instructed to avoid aerobic exercise during intervention.

#### 2.3.4. Anthropometry, Body Composition, and Dietary Intake Assessment

Body weight was measured using an electronic scale (Filizola PL 50, Filizzola Ltd., São Paulo, SP, Brazil). Height was measured on a fixed stadiometer (Sanny brand, São Paulo, SP, Brazil), and all the procedures were conducted according to the methods outlined by Rossi et al. [18]. A spectral bioelectrical impedance (BIA Analyzer, Nutritional Solutions, Harrisville, MI, USA) device was used to determine the total body water (TBW), intracellular water (ICW), extracellular water (ECW) content, resistance (R), and reactance (Xc). Participants were instructed to urinate before the measures, refrain from ingesting food or drink in the previous four hours, avoid strenuous physical exercise for at least 24 h, and avoid the consumption of alcoholic or caffeinated beverages for at least 48 h. Measurements were performed on a table that was isolated from electrical conductors, with subjects lying supine along the table’s longitudinal centerline axis, legs abducted at an angle of 45° relative to the body midline, and hands pronated. After cleaning the skin with alcohol, two electrodes were placed on the surface of the right hand, and two were placed on the right foot. The spectral bioelectrical impedance device was calibrated each day according to the manufacturer’s recommendations, and the exams were performed by the same professional in the pre- and post-intervention periods. The test–retest were measured 24 h apart and resulted in the following standard error of measurements (SEM) and intraclass correlation coefficients (ICC): SEM = 15.6 Ω and ICC = 0.95 for R, SEM = 3.5 Ω and ICC = 0.96 for Xc, (Souza et al.). The fat-free mass (FFM) was obtained from BIA prediction equations, according to the methods outlined by Wang et al. [25], and fat mass (FM in kg) was calculated (total body mass minus fat-free mass). All the measurements were performed at the same time of day (in the morning).

Food journals were distributed to all the participants to record food intake for three nonconsecutive days (one weekend and two weekdays), where the weekday corresponded to the day before the concurrent session. Participants were instructed by a nutritionist as to how to complete the dietary questionnaires. All the food intakes were analyzed for total kilocalorie and macronutrient intakes, and were averaged for the three days to ensure that dietary intake was similar between experimental trials. The software (Software-Dietpro Version 5.8, Viçosa, Minas Gerais, Brazil) utilized the database of the Brazilian food composition table (TACO) to calculate dietary intake.

#### 2.3.5. Supplementation Procedure

The participants consumed two 800-mg slow-release gel capsules (carboxymethylcellulose) in order to avoid paresthesia four times daily following meals, totaling 6.4 g β-alanine/day (Black Skull Supplement, São Paulo, SP, Brazil) or corresponding placebo capsules of maltodextrin in the same manner [19,21]. A 28-day supplementation period was chosen as the greatest increases in carnosine occur during the first four weeks of β-alanine supplementation [22]. The supplement packages were blinded so that neither the investigators nor the participants were aware of the contents until the completion of the analyses. To ensure that the participants took the supplements, participants received a one-week dose of capsules at the start of each week during the intervention. The subjects were instructed not to comment with other research subjects about possible side effects, and if there was any discomfort, they were asked to contact the nutritionist in the laboratory.

#### 2.3.6. Blood Lactate Concentration and Analysis

Participants stayed lying in a supine position for 10 min to start the blood sample collection. The blood sample was collected from the ear lobe to analyze the blood lactate concentration [Lac]. This measurement was obtained at seven time points, including: (1) at rest, (2) immediately post-HIIE, (3) pre-resistance exercise, (4) immediately post-resistance exercise, (5) 5 min post-resistance exercise, (6) 10 min post-resistance exercise, and (7) 30 min post-resistance exercise. The analyses were performed using the lactate analyzer Yellow Spring 1500 Sport (Yellow Springs, OH, USA).

#### 2.3.7. Statistical Analyses

Initially, we performed a power analysis of this study based on the observation from a previous study that verified the effect of β-alanine supplementation and resistance training on performance in untrained collegiate females. The authors verified a difference mean of 23.5 kg for maximum strength in leg press and the standard deviation of 11.1 kg after eight weeks of resistance training [18]. When we used a power (1-type II error) of 0.80 and a type I error of 0.05, via a formula suggested by Miot [26], it was estimated that we would need eight participants per group. Considering a percentage of dropout attrition in randomized controlled trials of around 32% [27], we over-recruited the number of participants. During the intervention, only one participant from the placebo group dropped out of the study due to unspecified reasons. After the intervention, there was an outlier in the β-alanine group, who was excluded from the final analysis. Thus, the final sample analyzed was 11 subjects in the placebo group and 11 in the β-alanine group.

Data are reported as means and standard deviation (SD). After normality assurance, a two-way variance analysis [group (β-alanine versus PLA) × time point (pre versus post)] was conducted to compare the maximal strength (absolute and relative to body mass), concurrent exercise acute volume (volume-load), *v*VO_2max_, rate of perceived exertion (RPE), body mass, fat-free mass, fat mass, TBW, ICW, ECW, R, and Xc. When a significant interaction effect was found, percent changes were calculated (∆% = post minus pre divided by pre multiplied by 100) and compared with an independent samples t-test. Statistical significance was set at *p* < 0.05. The effect sizes were calculated as the mean pre- to post-training change divided by the pooled pre-training standard deviation, whereby a value of >0.20 was considered small, >0.50 was considered moderate, and >0.80 was considered large. The data were analyzed using the Statistical Package for Social Sciences 17.0 (SPSS Inc., Chicago, IL, USA).

## 3. Results

The comparison between groups in the dietary intake are displayed in Table S2 in the Supplementary Material. There were no statistically significant differences between placebo and β-alanine supplementation at baseline for all the variables investigated.

### 3.1. Maximal Strength and Maximal Aerobic Velocity Results

Table 1 presents the maximal strength and maximal aerobic velocity pre and post 28 days of supplementation and strength training.

For absolute and relative to body mass maximal strength during the leg press, there was a main effect of time (F = 78.99; *p* < 0.001 [absolute values]; F = 69.76; *p* < 0.001 [relative to body mass]) with higher values performed post than pre training (*p* < 0.001), but no significant group x time interactions. There was no significant main effect of time (F = 0.161, *p* = 0.693), group x time interaction (F = 0.033, *p* = 0.857), and difference between groups (F = 2.222, *p* = 0.152) for *v*VO_2max_.

### 3.2. Acute Concurrent Exercise Results

Table 1 shows the pre- and post-intervention volume performed during four sets of leg presses at 80% 1 RM following HIIE.

For absolute and relative volume performed during the leg press (Table 1) there was a main effect of time (F = 54.05; *p* < 0.001 [absolute values]; F = 48.25; *p* < 0.001 [relative to body mass] with higher volume performed post- than pre-training (*p* < 0.001). There was also a main effect of time for RPE immediately after HIIE exercise (F = 6.28; *p* = 0.020), with lower scores post (16 ± 2 for β-alanine and 16 ± 2 for the placebo group) than pre-training (*p* = 0.020; 17 ± 2 for β-alanine and 17 ± 2 for the placebo group). There were no significant interactions for any of these variables.

**Figure 2:**
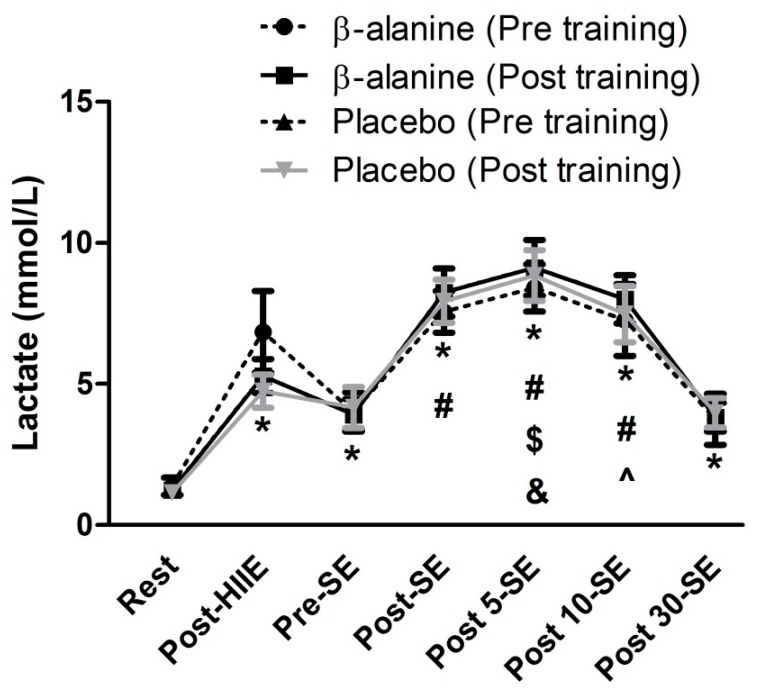
Blood lactate concentration in concurrent exercise session pre and post 28 days of supplementation combined with resistance training. Note: data are mean ± standard deviation. *: higher than rest; #: higher than pre-strength exercise (SE); $: higher than 30 min post-strength exercise; &: higher than 10 and 30 min post-strength exercise; ^: higher than 30 min post-strength exercise.

Figure 2 presents the blood lactate concentrations during the acute concurrent exercise session pre- and post-intervention. For lactate concentration (Figure 2), there was only a main effect of time (F = 272.53; *p* < 0.001) during the acute sessions, without any significant differences between pre- and post-intervention, nor any significant interactions.

### 3.3. Body Composition Results 

Table 1 presents the comparison between β-alanine and placebo on total body mass, fat-free mass, fat mass, total body water, extracellular water, and intracellular water.

For FFM, there was a trend for an interaction (F = 4.20; *p* = 0.053). When we compared percent changes, a non-significant trend was found for greater increases with β-alanine versus placebo [∆-β-alanine = 2.8 ± 1.8% (CI 95% 1.7 to 3.9) versus ∆-Placebo = 1.0 ± 2.5% (CI 95% −0.7 to 2.7%), *p* = 0.072]. There was also a main effect of time (F = 17.02; *p* < 0.001) with higher values post- than pre (*p* < 0.001). Effect sizes for FFM were small for β-alanine (0.30) and trivial for placebo (0.07). There was a main effect of time for body mass (F = 4.78; *p* = 0.040), ECW (F = 6.86; *p* = 0.019), ICW (F = 8.59; *p* = 0.010), and TBW (F = 6.02; *p* = 0.027), with higher values post- than pre-training for all. There was no main effect of time or significant differences between groups for fat mass and percentage of fat mass (*p* > 0.05).

### 3.4. Resistance Training Volume

Table 2 presents the total volume performed weekly during 28 days of supplementation combined with resistance training.

For volume performed each week during 28 days of supplementation (Table 2), there was only a main effect of time (F = 25.50; *p* < 0.001) with lower values in week 1 than weeks 2 (*p* < 0.001), 3 (*p* < 0.001), and 4 (*p* = 0.001); and week 2 was lower than week 4 (*p* = 0.011), but there were no significant group x time interaction (F = 1.318, *p* = 0.281). Effect sizes were large in all weeks for β-alanine and moderate to large in the placebo group.

## 4. Discussion

Our primary hypothesis was that 28 days of β-alanine supplementation would enhance acute resistance exercise performance by attenuating the reductions in volume that occur when resistance exercise is preceded by HIIE. Our results do not support this hypothesis, as both groups equally increased their leg press volume in the post-HIIE leg press test compared to pre-supplementation. We also did not find any differences between groups for total training volume or weekly increases in training volume.

To our knowledge, this was the first study to investigate the interaction between β-alanine supplementation and resistance exercise performance during acute concurrent exercise. Our results are very similar to a study recently published by Bassinello et al. [28]. In that study, resistance trained subjects were supplemented with 6.4 g/day of β-alanine during 28 days of non-structured resistance training. Compared to the placebo group, β-alanine supplementation did not increase repetition volume during eight sets of leg press to muscular failure with 70% 1 RM nor total work performed or fatigue index during five sets of isokinetic knee extension.

In contrast, Outlaw et al. [18] reported greater increases in leg press repetitions to failure following four and eight weeks of β-alanine supplementation. Differences in training status between Outlaw et al. [18] (untrained collegiate women) and the current study and Bassinello et al. [28] (resistance trained men) and the training period, as well as the supplementation period (eight weeks versus 28 days) may explain these discrepancies in results. Additionally, given that females tend to have less intramuscular carnosine concentrations when matched for aged and training status compared to males [29], gender differences may also influence the ergogenic effects of β-alanine supplementation. Two other studies have investigated the effects of β-alanine on training volume or repetitions to fatigue. In the first study, Hoffman et al. [30] reported that the addition of β-alanine to creatine increased resistance exercise capacity greater than creatine alone. However, a β-alanine only group was not employed in this study, making it difficult to isolate the ergogenic effect of β-alanine. In a second study, Hoffman et al. [31] reported that β-alanine supplementation increased repetitions to fatigue during back squats with 70% 1 RM. This study utilized a repeated measures design; however, as discussed by Bassinello et al. [28], the washout period was only four weeks, which was likely not sufficient to allow for carnosine concentrations to return to baseline [32].

Beta-alanine has been shown to reduce skeletal muscle fatigue by increasing the muscle buffering capacity during maximal high-intensity exercises [33]. As such, our primary hypothesis was that β-alanine would enhance resistance exercise performance when preceded by HIIE via enhanced H^+^ buffering. Although we were unable to measure blood pH or H^+^ concentrations, using a similar resistance exercise protocol, Bassinello et al. [28] reported reductions in plasma pH (pH = 7.25 ± 0.03) following just two sets of leg press to failure with 2 min of rest between sets, without further reductions in pH occurring in sets four to eight. Therefore, it is likely that the exercise protocol used herein generated an intracellular metabolic environment whereby increased intramuscular carnosine should enhance performance via increased buffering. Not only were there no differences in performance between groups, but there were also no differences between groups or across time (pre- versus post-supplementation) for lactate. These results suggest that, at least in the context of performing the leg press exercise when preceded by HIIE, intracellular acidosis was not a major contributor to fatigue, and β-alanine supplementation does not support greater increases in resistance training volume with moderate (70–80% 1 RM) loads. Supporting this, Morales-Alamo et al. [34] examined if muscle metabolites cause task failure during incremental exercise to exhaustion in conditions of normoxia and hypoxia in healthy men. The results showed that during incremental exercise, task failure is not due to metabolite accumulation (lactate and H^+^) or lack of energy resources. These findings lead us to hypothesize that central mechanisms or mechanisms not related to metabolite accumulation mediated fatigue during the leg press exercise when preceded by HIIE.

The second hypothesis of the present investigation was that β-alanine would potentiate increases in lean mass when combined with resistance training. We could not confirm this hypothesis, even though there was a tendency for greater increases in FFM in the β-alanine group (β-alanine = 2.8% versus placebo = 1.0%), post hoc testing did not reach statistical significance, and the effect size for the β-alanine group was small (*d* = 0.30). These results support our contention that the ability of β-alanine supplementation enhance resistance training and thereby muscle hypertrophy is unclear, and agrees with the mixed results reported in the literature, as some [17,35], but not all [28,36] studies report greater improvements in lean mass.

Two possible hypotheses have been proposed to explain the lean mass gains reported with β-alanine supplementation. First, since greater resistance training volumes are highly associated with muscular hypertrophy outcomes [37], we hypothesized that subjects in the β-alanine group could sustain more prolonged high-intensity muscular contractions by increasing muscle buffering capacity and calcium sensitivity in muscle fibers. These adaptations could result in the generation of increased total training volume, thereby possibly resulting in greater metabolic stress (via lactate accumulation) and cellular swelling, which may indirectly [16] or directly promote increased muscle hypertrophy [38] or muscle regeneration [39]. Our results do not support this hypothesis, as there were no differences in volume accomplished or lactate concentrations during the acute concurrent training test between groups.

Secondly, there were no significant differences in weekly increases in volume between groups. It is possible that a longer period of training would result in more pronounced differences between groups, as Kern et al. [17] reported significant differences following eight weeks of training. On the other hand, Smith et al. [35] reported significant increases in lean mass following three weeks of training and supplementation, with no differences between weeks three and six.

Fluid shifts into the muscle and increases in intracellular water may be responsible for the early onset of muscle hypertrophy, and have been proposed to account for some of the increases in lean mass seen with β-alanine supplementation [17]. Although we were not able to measure the accumulation of myofibrillar protein content in the exercised muscles, and although there were no statistically significant differences between groups for total or intracellular water content, the effects sizes favored the β-alanine group for both variables (0.26 versus 0.08 and 0.62 versus 0.40, respectively). β-alanine acts as an intracellular osmolyte and increases intracellular water content in vitro, lending support to this hypothesis [40], and possibly explaining the small—but non-significant—increase in lean mass found in the supplemented group.

Beta-alanine supplementation also did not enhance strength adaptations to resistance training in this study compared to the placebo group. These results agree with those of Outlaw et al. [18], whereby eight weeks of β-alanine supplementation (3.4 g/day for eight weeks) did not increase maximum strength in the leg press or bench press. In contrast, Maté-Muñoz et al. [41] reported greater increases in maximal strength and power following five weeks of β-alanine supplementation. Whether β-alanine supplementation benefits maximal strength adaptations associated with resistance training still requires further investigation [36].

Despite the importance of our data, it is necessary mention the limitations of this study. First, a lack of metabolic analysis, muscle buffering capacity, muscle carnosine concentration, and calcium release prevent us from drawing any mechanistic conclusions. Second, in the present study, the subjects did not perform concurrent training during 28 days, as the main objective was to analyze the acute interference effects induced by HIIE. We suggested future researchers investigate the effect of β-alanine on chronic concurrent training and verify longer periods of intervention.

## 5. Conclusions

In conclusion, 28 days of β-alanine supplementation did not prevent acute strength loss during resistance exercise after high-intensity intermittent exercise, nor increased strength or hypertrophic adaptations associated with resistance training.

## Figures and Tables

**Table 1:** Body composition, maximal strength, maximal aerobic velocity, and volume performed in the leg-press in four sets (at 80% of maximal strength), after high-intensity intermittent exercise pre and post 28 days of supplementation and strength training.

Variables	Group	Pre	Post	Effect Size
Maximal strength (kg)	β-alanine	340 ± 61	410 ± 71 *	1.05
	Placebo	317 ± 67	368 ± 77 *	0.70
Maximal strength relative to body mass (kg/kg)	β-alanine	4.2 ± 0.5	5.1 ± 0.6 *	1.63
	Placebo	4.1 ± 0.6	4.8 ± 0.7 *	1.08
Maximal aerobic velocity (km/h)	β-alanine	13.9 ± 1.0	13.9 ± 1.1	0.00
	Placebo	13.2 ± 1.1	13.3 ± 1.0	0.10
Volume performed in leg press (kg)	β-alanine	8754 ± 4179	11922 ± 3494 *	0.82
	Placebo	7376 ± 3394	10953 ± 4162 *	0.95
Volume performed in leg press relative to body mass (kg/kg)	β-alanine	107.2 ± 46.1	145.6 ± 33.6 *	0.95
	Placebo	96.2 ± 37.7	142.9 ± 46.3 *	1.11
Body mass (kg)	β-alanine	80.1 ± 8.0	80.8 ± 8.1 *	0.08
	Placebo	77.3 ± 15.8	77.5 ± 15.4 *	0.01
Fat-free mass (kg)	β-alanine	64.0 ± 5.7	65.8 ± 6.3 *	0.30
	Placebo	62.4 ± 9.2	63.1 ± 10.0 *	0.07
Fat mass (kg)	β-alanine	19.8 ± 6.5	19.5 ± 7.5	0.04
	Placebo	19.6 ± 11.9	19.3 ± 11.7	0.03
Body fat (%)	β-alanine	18.9 ± 5.7	17.8 ± 5.1	−0.20
	Placebo	17.3 ± 6.8	19.3 ± 11.7	0.22
Extracellular water (L)	β-alanine	18.0 ± 1.8	18.6 ± 2.0 *	0.32
	Placebo	17.9 ± 2.7	18.1 ± 3.1 *	0.07
Intracellular water (L)	β-alanine	28.3 ± 2.9	29.1 ± 3.2 *	0.26
	Placebo	28.2 ± 4.7	28.4 ± 5.3 *	0.04
Total body water (L)	β-alanine	46.4 ± 4.8	47.7 ± 5.2 *	0.26
	Placebo	44.8 ± 6.7	45.0 ± 7.6 *	0.03
Resistance (ohms)	β-alanine	457.1 ± 32.9	449.7 ± 33.3	0.24
	Placebo	477.1 ± 56.4	470.9 ± 70.8	0.10
Reactance (ohms)	β-alanine	52.3 ± 6.4	51.1 ± 8.0	0.16
	Placebo	53.8 ± 6.3	52.2 ± 7.1	0.24

Note: Data are mean ± standard deviation; *: higher than pre values (*p* < 0.05).

**Table 2:** Total volume performed weekly during 28 days of supplementation combined with resistance training.

Period	Group	Total Weekly Volume (kg)	Effect Size
Week 1 *	Beta	27.048 ± 5582	NA
	Placebo	24.499 ± 6053	NA
Week 2 #	Beta	32.705 ± 5484	1.02
	Placebo	27.958 ± 5759	0.59
Week 3	Beta	33.521 ± 5386	1.18
	Placebo	29.370 ± 5871	0.82
Week 4	Beta	35.323 ± 5631	1.48
	Placebo	29.574 ± 5283	0.90

Note: Data are mean ± standard deviation. *: Lower than weeks 2, 3, and 4 (*p* < 0.05); #: Lower than week 4 (*p* < 0.05). Effect size: Week 2, week 3, and week 4 in relation to week 1. NA = not applicable.

## Supplementary Materials

The following are available online at https://www.mdpi.com/2075-4663/7/5/108/s1, Table S1: Resistance training program, Table S2: Comparison between placebo and β-alanine on the dietary intake and macronutrient distribution.
