# Supplementary material for: Short-Time β-Alanine Supplementation on the Acute Strength Performance after High-Intensity Intermittent Exercise in Recreationally Trained Men
Source: Sports (Basel). 2019 May 9;7(5):108. doi: 10.3390/sports7050108 (PMC6571921; doi:10.3390/sports7050108)
Supplement: Supplementary file 1 [file sports-07-00108-s001.pdf]

sports

Supplementary Information

Short-Time β-Alanine Supplementation on the Acute Strength Performance after High-Intensity Intermittent Exercise in Recreationally Trained Men

Marcelo Conrado Freitas, Jason Cholewa, Valéria Panissa, Giovanni Quizzini, João Vitor de Oliveira, Caique Figueiredo, Luis Alberto Gobbo, Erico Caperuto, Nelo Eidy Zanchi, Fabio Lira and Fabrício Eduardo Rossi

Table 1. Resistance training program.

	Movement	Sets	Repetition
Day 1	Bench Press	3	10–12
	Incline Dumbbell Press	3	10–12
	Peck Deck	3	10–12
	Dumbbell Overhead	3	10–12
	Lateral Dumbbell Raises	3	10–12
	Lying Triceps Extension	3	10–12
	Triceps Press Down	3	10–12
Day 2	Back Squat	3	10–12
	45° Leg Press	3	10–12
	Leg Extensions	3	10–12
	Calf Raises	3	10–12
	Crunches	3	15–20
	Hanging Leg Raises	3	15–20
Day 3	Lat Pulldown	3	10–12
	T-Bar Row	3	10–12
	Cable Row	3	10–12
	Barbell Curls	3	10–12
	Scott Curls	3	10–12
Day 4	Traveling Lunges	3	10–12
	Stiff leg deadlifts	3	10–12
	Leg Curls	3	10–12
	Calf Raises	3	10–12
	Crunches	3	15–20
	Hanging Leg Raises	3	15–20

Table 2. Comparison between placebo and β-alanine on the dietary intake and macronutrient distribution.

Variables	Placebo (n=11)		β-alanine (n=11)		Time x Group
**Dietary Intake**	**Pre**	**Post**	**Pre**	**Post**	
Carbohydrate (g)	264.8 ± 107.8	315.5 ± 176.6	261.5 ± 149.7	249.4 ± 127.9	0.214
Carbohydrate (g/body weight)	3.9 ± 2.2	4.5 ± 2.8	3.3 ± 1.7	3.1 ± 1.6	0.213
Protein (g)	99.4 ± 32	111.6 ± 25.7	128.6 ± 38.4	143.6 ± 44.5	0.815
Protein (g/body weight)	1.4 ± 0.5	1.6 ± 0.5	1.6 ± 0.4	1.8 ± 0.6	0.817
Lipids (g)	72.7 ± 18.6	88.8 ± 33.1	65.7 ± 20.7	76.7 ± 29.7	0.693
Lipids (g/body weight)	1 ± 0.3	1.2 ± 0.4	0.8 ± 0.3	1 ± 0.4	0.620
Total Intake (kcal)	2123.2 ± 620.9	2513.4 ± 904.7	2209.5 ± 504.8	2277.0 ± 650.5	0.245
Total Intake (kcal/body weight)	31.1 ± 13.3	35.9 ± 15.6	28.3 ± 6.5	29.3 ± 10	0.297
